# Structural Characterization of Quinoa Polysaccharide and Its Inhibitory Effects on 3T3-L1 Adipocyte Differentiation

**DOI:** 10.3390/foods9101511

**Published:** 2020-10-21

**Authors:** Cong Teng, Zhenxing Shi, Yang Yao, Guixing Ren

**Affiliations:** 1 Institute of Crop Science, Chinese Academy of Agricultural Sciences, Beijing 100081, China; 82101172124@caas.cn (C.T.); shizhengxing@caas.cn (Z.S.); renguixing@caas.cn (G.R.); 2 Laboratory of Biomass and Green Technologies, Gembloux Agro-Bio Tech, University of Liège, 5030 Gembloux, Belgium

**Keywords:** polysaccharide purification, anti-obesity, proliferation, PPARγ

## Abstract

Quinoa is a kind of nutritious food crop with anti-obesity activity, however, the mechanism is not unclear. In this study, we separated and purified bioactive polysaccharide from quinoa (denoted SQWP-2). The chemical structural was characterized and its effect on 3T3-L1 pre-adipocyte differentiation was evaluated. The molecular weight of SQWP-2 was found to be 7.49 × 10^3^ Da, and the polysaccharide consisted of fructose and glucose. The Glc-(1→, Fru-(2→, →4)-Glcp-(1→, and →4,6)-Glcp-(1→ glycosidic linkages were identified in SQWP-2 through gas chromatography-mass spectrometry. Nuclear magnetic resonance confirmed the monosaccharide composition and glycosidic linkage content, and a suggestion of the structural formula is provided. In Western Blotting and RT-PCR assays, treatment with SQWP-2 significantly inhibited 3T3-L1 differentiation by suppressing PPARγ, C/EBPα, C/EBPβ, C/EBPδ, SREBP1C and AP2 expression. Quinoa polysaccharide isolated here could represent an anti-obesity agent once the structures and differentiation inhibition are definitively characterized.

## 1. Introduction

Obesity is a complex health disorder caused by the accumulation of adipose tissue due to increasing and enlarged fat cells [1]. Obesity is related not only to higher mortality, but also to an increased risk of cardiovascular disease, diabetes, gallbladder disease, and various cancers [2]. Therefore, finding the food trophic factors that can inhibit the condition is of high importance. As a molecular mechanism for adipogenesis, 3T3-L1 cell differentiation efficiency is an important factor associated with obesity and related diseases. This process is currently the subject of considerable research and is widely used as an adipocyte differentiation model system [3,4]. 

Quinoa is an important food crop, which can meet the demands of human basic nutrition according to the United Nations of Food and Agriculture Organization [5,6]. It is beneficial for human health owing to its density of nutrients including amino acids, minerals, phytochemicals, and active polysaccharides [7,8]. Previous research has demonstrated that quinoa has a significant anti-obesity effect. Farinazzi-Machado et al. reported that 30 days of consumption of quinoa candies resulted in significant reductions in body weight as well as triglycerides (TGs) and low-density lipoprotein (LDL) levels among 22 students aged 18–45 [9]. The components of quinoa identified as inducing the anti-obesity effect of the product were saponin, 20-hydroxyecdysone, and dietary fiber [9,10,11]. Oil red O staining and intracellular quantitation analyses confirmed that saponin from quinoa inhibits the accumulation of TG in mature adipocytes and significantly inhibits the expression of peroxisome proliferator-activated receptor gamma (PPARγ) and CCAAT/enhancer-binding protein alpha (C/EBPα), which are [9] key transcription factors for messenger RNA expression and protein fat formation. Compared with high-fat (HF) mice, treated mice exhibited significantly lower mRNA levels of several markers of inflammation and insulin resistance, Foucault observed that administration of 20-hydroxyecdysone from quinoa could prevent diet-induced obesity and regulate adipocyte-specific gene expression in mice [10]. In Maha’s research, the administration of quinoa dietary fiber was found to reduce levels of cholesterol components (LDL and high-density lipoprotein), TGs, and total lipids in rats [11]. Many plant polysaccharides have been reported to inhibit 3T3-L1 adipocyte differentiation within cell systems [12]. Polysaccharides from pine needles have been reported to have an anti-lipogenesis effect via regulating lipid metabolism genes encoding transcription factors and cytokines [13]. In Zhu’s research, barley β-glucan was found to inhibit adipocyte differentiation by causing downregulation of PPARγ, C/EBPα, and Glut4 mRNA and protein expression levels [14]. Recent research has also shown that certain polysaccharides from quinoa possess antioxidant, immunoregulatory, and anticancer activities [15]. However, the anti-obesity activities of quinoa polysaccharides have not yet been studied.

Macromolecular activity is related to chemical structure [16]. Ferreira found that the immunomodulatory activity of polysaccharides main attributed to the proportion of glycosidic linkages [17]. However, the structure of quinoa polysaccharides is not entirely clear, and the exact relationship between structure and activity of quinoa polysaccharides has not been covered.

The purpose of this study was to (1) characterize the structure of quinoa polysaccharide, (2) evaluate the inhibitory effects of quinoa polysaccharide on 3T3-L1 adipocyte differentiation in vitro.

## 2. Materials and Methods

### 2.1. Plant Sample and Reagents

Quinoa seeds (Cultivar MengLi-1) were obtained from the Chinese Academy of Agricultural Sciences, Beijing, China. The seeds were crushed [9] and sifting through a 600-μm sieve, then stored in a freezer until use, not more than 15 days after grinding.

Diethylaminoethyl (DEAE)-Sepharose Fast Flow resin was purchased from GE Healthcare Life Sciences (Uppsala, Sweden), and 3T3-L1 cells were obtained from the Institute for Biological Sciences, Chinese Academy of Sciences, Shanghai, China. Dulbecco’s modified Eagle’s medium (DMEM), insulin, Fetal bovine serum (FBS), dexamethasone (DEX), 1-methyl-3-isobutylxanthine (IBMX) and a-amylase were purchased from Sigma Chemical Co. (St. Louis, MO, USA).

### 2.2. Extraction and Purification

The prepared sample was extracted using 95% ethanol at a ratio of 1:8 for 3 h following centrifugation (3000× *g*, 10 min). The 500 g defatted quinoa powder was collected and extracted twice using distilled water (90 °C, 4 h) at a ratio of 1:10. After centrifugation (4000× *g*, 10 min), the supernatant was collected and concentrated. Chloroform-n-butanol was used to deproteinize according to the Sevage method. To remove starch, fractions were sufficiently treated with α-amylase and dialyzed [18]. A polysaccharide fraction was obtained, which was then purified by chromatography using an ÄKTA Explore 100 purification system (General Electric, Stockholm, Sweden). The polysaccharide was dissolved in a Tris-HCl buffer solution at pH 7.5 with 0.1 mM CaCl_2_, 2.5 mM MgCl_2_, and 0.06% NaN_3_ was added and stirred well, centrifuged (13,000× *g*, 10 min), after which supernatant was uploaded onto a DEAE Sepharose Fast Flow column (2.6 cm × 100 cm). Fractions collected according to absorbance detected by phenol—sulfuric acid method were further purified on a Sephacryl S-300 high-resolution column (Dextran separation range 2 × 10^3^–4 × 10^5^) [19]. Finally, 36.1 g SQWP-2 was obtained from defatted quinoa powder. The polysaccharide purity was tested using HPLC-ELSD method equipped with a Shodex Asahipak NH2P-50 4E column (250 mm × 4.6 mm × 5 μm, Agilent Technologies, Santa Clara, CA, USA). A solution containing acetonitrile and water (4:1) was used as the mobile phase at a flow rate of 1.0 mL min^−1^_,_ and the column oven temperature was 35 ℃. Drift tube temperature was 70 °C. Spray tube temperature was 30 °C. The carrier gas flow rate was 1.1 mL min^−1^.

### 2.3. Evaluation of the Structure Characteristics of the Water-Soluble Polysaccharide

#### 2.3.1. Scanning Electron Microscopy

Purified bioactive polysaccharide from quinoa (denoted SQWP-2) was examined using a Sigma 300 scanning electron microscope (SEM, AMICS, Berlin, Germany). Mica surfaces were used, and 1 cm^2^ of tape removed to expose fresh surfaces. The SQWP-2 polysaccharide powder was placed on the mica slices and samples imaged at 100- and 1000-times magnification. The surface morphology of samples was mainly revealed by secondary electron signal imaging [20].

#### 2.3.2. Molecular Weight Determination

Size exclusion chromatography was used to measure the average molecular weight (Mw) performed using two PL aquagel-OH Mixed 8-μm columns (Tosoh Bioscience GmbH, Griesheim, Germany: 300 × 7.5 mm) with a PL aquagel-OH guard protection 8-μm precolumn for gel permeation chromatography (GPC). Eluent (0.1 M NaNO_3_ solution) was pumped at a flow rate of 0.9 mL/min. The column was calibrated in the range of 5.8–1600 kDa using Pullan standards (British Polymer Laboratory).

#### 2.3.3. Fourier-Transform Infrared Spectroscopy

Infrared (IR) spectroscopy was performed using a Fourier-transform IR system (Bruker, Rheinstetten, Germany) with a scanning range of 4000–500 cm^−1^.

#### 2.3.4. Monosaccharide Composition Analysis

Acetylated derivatives were prepared by hydrolysis, reduction, and acetylation of polysaccharides for analysis using previously published methods [21]. Briefly, The RXI-5 SIL MS column (30 m × 0.25 mm × 0.25 mm) was used for gas chromatograph-mass spectrometry (GC-MS), with an initial temperature of 120 °C, final temperature of 250 °C at a flow rate of 1 mL min^−1^.

#### 2.3.5. Analysis of Glycosidic Linkages

Glycosidic linkages were determined by GC-MS according to the published method [22]. The sample (2 mg) was hydrolyzed with 1 mL trifluoroacetic acid (2 mol L^−1^) for 90 min and then the residues were dissolved into double distilled water (2 mL) and NaBH_4_ (100 mg), respectively. One hundred microliters of glacial acetic acid was added after the reduction. The sample was dried under reduced pressure, and then acetylated with 1.0 mL acetic anhydride at 100 °C for 1 h. The acetylated derivatives were extracted with 3 mL chloroform and washed with water. The analysis was performed using a Shimadzu GCMS-QP 2010 gas chromatography-mass spectrometer.

#### 2.3.6. Nuclear Magnetic Resonance

The ^1^H and ^13^C spectra and DEPT135, HSQC, HMBC, NOESY spectra of the SQWP-2 were recorded at 30 °C with AV-500 MHz spectrometer (Bruker, Rheinstetten, German). Tetramethylsilane was used as an internal standard.

### 2.4. Evaluation of the Inhibition of Adipocyte Differentiation by the Water-Soluble Polysaccharide

#### 2.4.1. Cell Culture

3T3-L1 cells were cultured according to the suggested protocol from the Chinese Academy of Sciences Shanghai Cell Bank (Shanghai, China). Cells grew in DMEM supplemented with 4.5 g L^−1^ D-glucose, 10% FBS, and 1% penicillin at 37 °C in an atmosphere containing 5% CO_2_. 

#### 2.4.2. Viability Assay

Cell viability was evaluated using the 3-(4, 5-dimethylthiazol-2-yl)-2, 5-diphenyltetrazolium bromide (MTT) method [23]. Briefly, cells were distributed into 96-well plates (2 × 10^5^ cells mL^−1^) and cultivated overnight (37 °C, 5% CO_2_). After 24 h, DMEM cell medium containing SQWP-2 (0.5, 1, 2, 4, 8 mg ml−1) was added to the treated groups. Cells were cultivated for a further 24 h, after that 20 μL of MTT was added to the treated and control groups and incubation continued for 4 h. Then the MTT reagent was removed, sulfoxide (DMSO) was added (150 μL/well), and the mixture was shaken for 15 min to promote the dissolution of the purple crystals. The OD value was measured at 570 nm.

#### 2.4.3. Cell Differentiation Assay

We placed 2 mL of 3T3-L1 pre-adipocytes into each well of a 12-well plate (2.5 × 10^5^ cells mL^−1^) and cultured the cells in a treated DMEM cell medium. Cells were grown to fusion at 37 °C with 5% CO_2_ [24]. After fusion for 48 h, cell contact was prevented (this time was defined as differentiation day 0). Cells were cultured in DMEM for 48 h (day 2), then medium replaced with DMEM containing 5 mg L^−1^ of insulin and 10% fetal calf serum, cultured for a further 48 h (day 4). After that, the medium was changed every 48 h (using DMEM with 10% FBS), until approximately 90% of the pre-adipocytes were differentiated into adipocytes. The fat content was assessed on day 8. 

#### 2.4.4. Oil-Red O Staining and Measurement of Optical Density

Differentiation of the cell cultures described in Section 2.4.3 was induced on day 8. The culture solution was removed and cells were fixed with 4% paraformaldehyde for 30 min. Oil red O stain solution was added. After staining for 60 min, the solution was removed and replaced with phosphate-buffered saline (PBS). Cells were observed and photographed under an inverted microscope (Thermo Fisher Scientific, Waltham, MA, USA), then 200 μL of isopropyl alcohol added to fully dissolve oil red O in stained adipocytes and the OD measured at 492 nm. 

#### 2.4.5. RNA Extraction and Real-Time Reverse Transcription Polymerase Chain Reaction

Total mRNA was extracted from 3T3-L1 cells collected on day 8 using the Trizol Reagent (solarbio, Beijing, China). Total RNA was transcribed into complementary DNA (cDNA) using a large-capacity cDNA reverse transcription kit (Sangon, Shanghai, China). Gene expression was quantitatively analyzed by real-time polymerase chain reaction (PCR) using a TaqMan Fast Universal PCR Master Mix (Applied Biosystems) Primers used for PCR are shown in Table 1. The 2^−ΔΔCT^ method was used to calculate the relative expression of each gene using β-actin as an internal standard. The relative levels of gene transcripts following exposure to SQWP-2 are expressed as fold change. Experiments were carried out in triplicate.

#### 2.4.6. Western Blot Analysis

Western blot analysis was performed according to the published method [14]. After incubated with 4 mg mL^−1^ SQWP-2 for 24 h, 3T3-L1 cells incubated in lysis buffer (Sangon, Shanghai, China) on ice for 20 min. Cytolyte supernatant was collected and protein content was estimated after centrifugation at 10,000× *g* (4 °C, 20 min). Protein samples (10 μg) were separated using 10% sodium dodecyl sulfate-polyacrylamide gel electrophoresis (SDS-PAGE) and transferred to polyvinylidene fluoride (PVDF) membranes. After blocking with 5% skim milk in Tris-buffer salt containing 0.1% Tween-20 (TBST) for 1 h, anti-PPARγ, anti-C/EBPα, anti-CCAAT/enhancer-binding protein beta (C/EBPβ), anti-CCAAT/enhancer-binding protein delta(C/EBPδ), anti-Sterol regulatory element-binding protein-1c (SREBP1C) and anti-adipocyte protein 2 (AP2) and anti-β-actin antibodies (Sangon, Shanghai, China) were added in combinations and the cells were incubated for 2 h at room temperature and washed with TBST. Horseradish peroxidase (HRP)-labeled secondary antibodies were added for 1 h, after which cells were washed with TBST for 10 min. Signals were detected by ELISA Pico chemiluminescent substrate (Sangon, Shanghai, China).

#### 2.4.7. Statistical Analysis

We used SPSS V.13 (SPSS Inc., Chicago, IL, USA) for all statistical analyses. One-way analysis of variance (ANOVA) and Duncan’s New Multiple-Range test was used to assess statistical differences between groups. Differences were considered statistically significant at *p* < 0.05.

## 3. Results

### 3.1. Extraction and Purification

**Figure 1:**
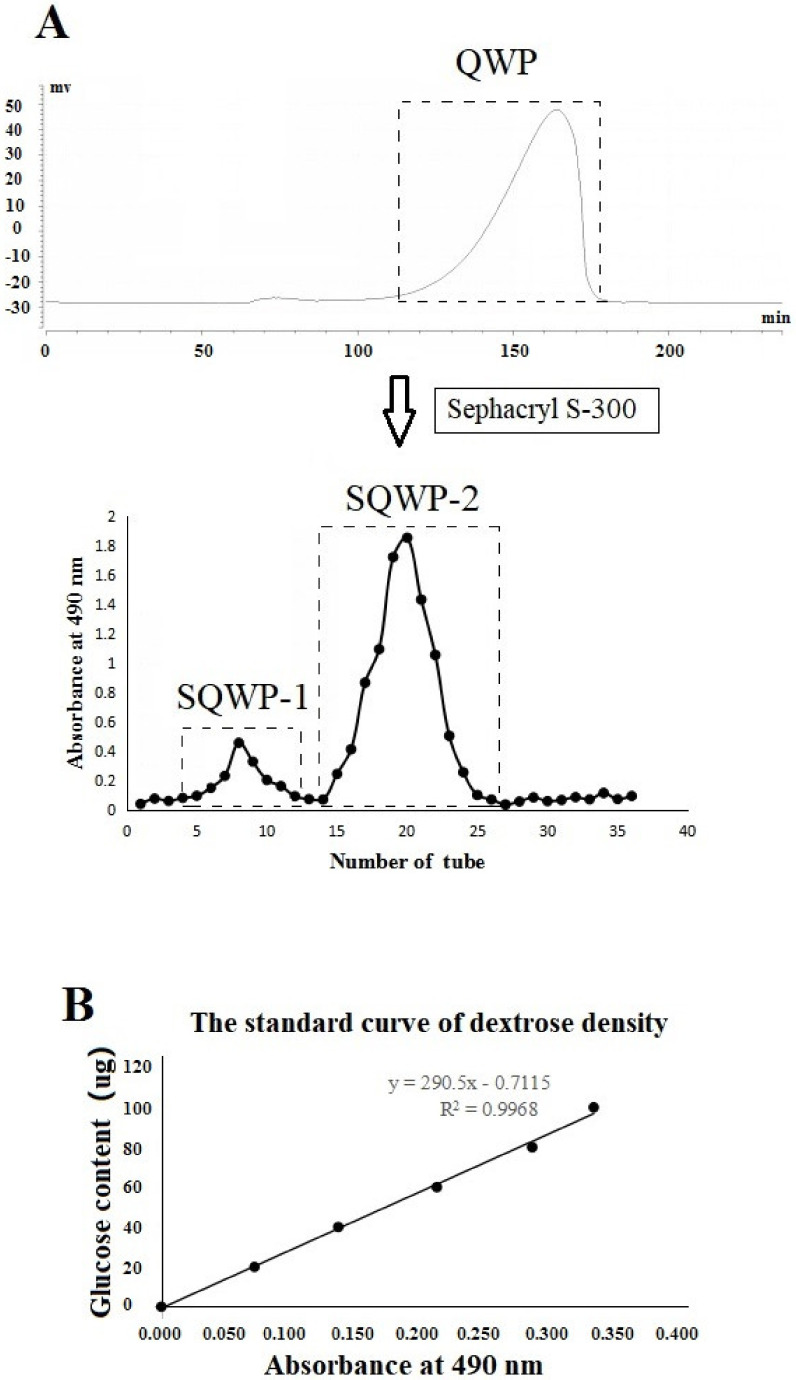
(**A**) Elution curve of the water-extractable (SQWP-2) on a Sepharose Flast Flow column and gel filtration chromatography; (**B**) standard curve of dextrose density.

The purity of QWP isolated from quinoa seeds was approximately 64.1%. Chromatographic purification enables the required fractions to be collected (Figure 1). After purification, the purity of SQWP-2 was up to 95%. In the pre-experiment, the SQWP-2 showed a higher inhibitory effect on 3T3-L1 cell differentiation than SQWP-1 (Figure S1). Therefore, SQWP-2 was further analyzed its chemical structure and reveal the mechanism of its anti-adipogenesis effect.

### 3.2. Analysis of Surface Morphology

**Figure 2:**
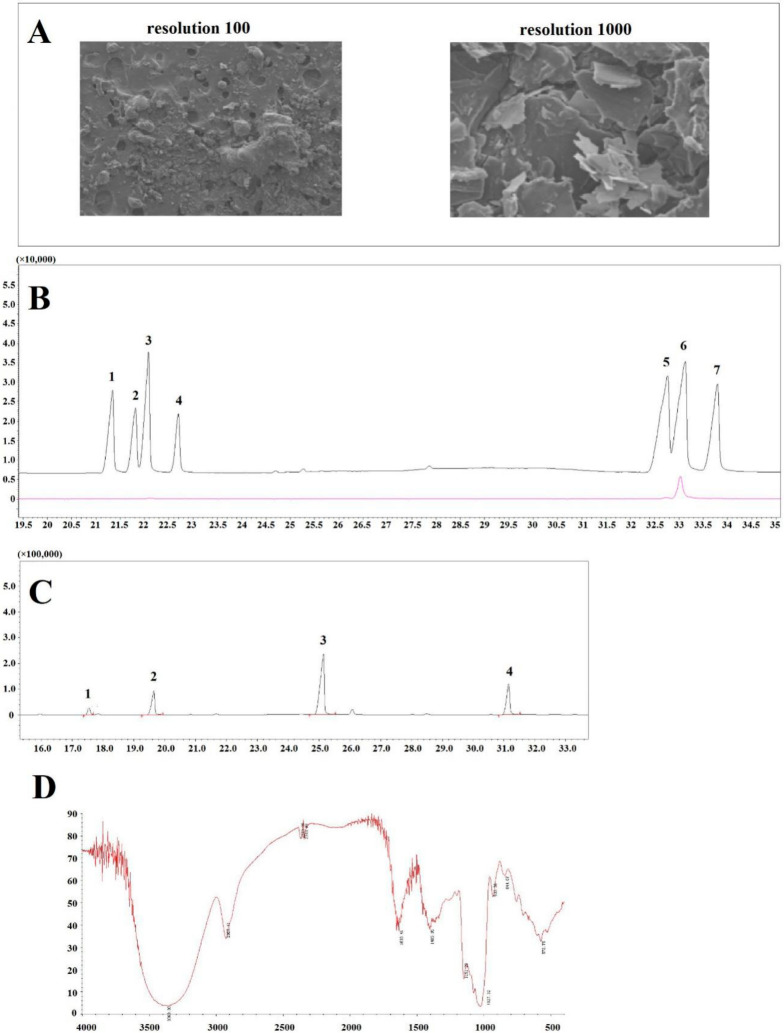
(**A**) Scanning electron microscopy (A × 100, B × 1000); (**B**) monosaccharide composition analysis by chromatograph-mass spectrometry (GC-MS). (1). Rham, (2). Fuc, (3). Ara, (4). Xyl, (5). Man, (6). Glu, (7). Gal; (**C**) Glycosidic linkage by methylation analysis. (1). 1,3,4,6-Me4-Glc/Manp, (2). 2,3,4,6-Me4-Glcp, (3). 2,3,6-Me3-Glcp, (4). 2,3-Me2-Glcp; (**D**) FT-IR spectra of SQWP-2.

A representative SEM micrograph of SQWP-2 is shown in Figure 2A, SQWP-2 appeared globular in the structure at 100-times magnification. At 1000-times magnification, the surface of the polysaccharide appeared rough and dentate.

### 3.3. Analysis of Monosaccharide Composition and Glycosidic Linkages

Glycosidic linkage of SQWP-2 was analyzed by GC/MS. Gas chromatography analysis indicated SQWP-2 to be composed of glucose (Figure 2B) and methylation analysis revealed the presence of four types of glycosidic linkage: 2,3,4,6-Me4-Glcp; 1,3,4,6-Me4-Glc/Manp; 2,3,6-Me3-Glcp; and 2,3-Me2-Glcp (Figure 2C). Their corresponding link way for Glc-(1→, Fru-(2→, →4)-Glcp-(1→, →4,6)-Glcp-(1→ is detailed in Table 2. The polysaccharide was found to be composed of dextran, whose main chain is →4)-Glcp-(1→ and exhibits *O-*6 branching.

### 3.4. Fourier-Transform Infrared Spectroscopy

As Figure 2D showed, an absorption band at 3600–3200 cm^−1^ was observed, which could be attributed to the stretching vibration absorption peak of –OH. The peak at 2927 cm^−1^ relates to the C–H stretching vibration of polysaccharides. An absorption peak was noted at 1633 cm^−1^ due to the asymmetric stretching vibration of C=O. Peak at 1405 cm^−1^ could be attributed to C–H variable angular vibration. The absorption peaks between 1020 cm^−1^ and 1160 cm^−1^ were due to the C–O stretching vibration and the peak at 844 cm^−1^ may be attributed to alpha-terminal radical isomerism. The specific structure of the polysaccharide needs to be confirmed by further nuclear magnetism analysis.

### 3.5. Nuclear Magnetic Spectroscopy

**Figure 3:**
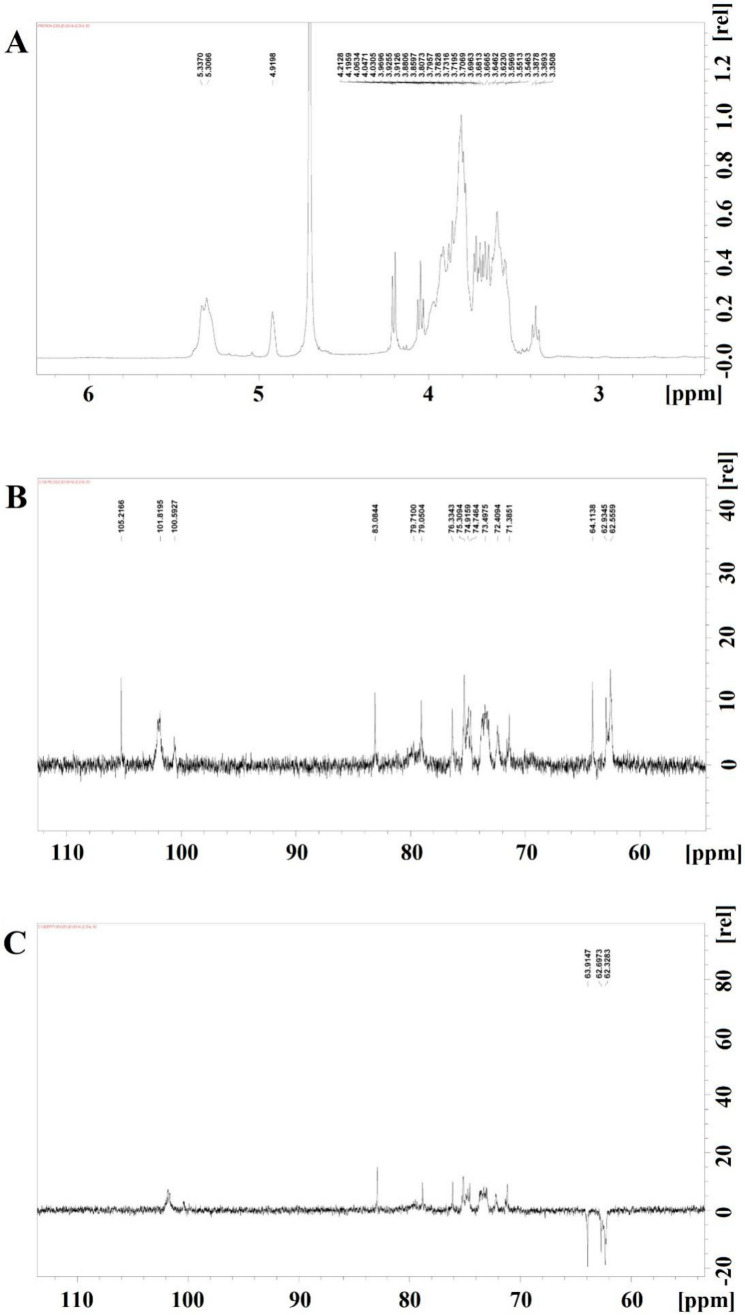
The nuclear magnetic resonance (NMR) spectra analysis of SQWP-2. (**A**) ^1^HNMR; (**B**) ^13^C NMR; (**C**) Dept135.

The ^1^H-nuclear magnetic resonance (NMR), ^13^C-NMR, DEPT135 of SQWP-2 were shown in Figure 3A–C, respectively. The δ 3.2–4.0 ppm signal in the hydrogen spectrum related to the proton of sugar-ring. The ^13^C NMR spectrum (126 MHz, D_2_O) signals were observed to be mainly concentrated between 60 and 120 ppm. Sharp signals at 100.59, 101.82, and 105.21 ppm indicated an α configuration of the glycosidic linkages, and signals were observed in the range of δ 60–85 indicating the presence of (1→4)-α-glycosidic linkages. The results indicated that the analyzed polysaccharides were mainly composed of dextran with a small amount of fructose. The DEPT135 chromatogram revealed inverted peaks at 62.32, 62.70, and 63.91 ppm, indicating the chemical displacement of the C6 or C1 signal peak of fructose or CH_2_ groups of either glucose or fructose.

Because the area of an absorption peak is proportional to the number of hydrogen protons, H1 of →4)-α-D-Glcp-(1→, →4,6)-α-D-Glcp-(1→, α-D-Glcp-1→, and H3 of β-D-Fru-(2→ were integrated, which were found to exist in a ratio of 3.5:1:1.4. The corresponding ratio of hydrogen protons for →4)-α-D-Glcp- (1→, →4,6)-α-D-Glcp-(1→ including α-D-Glcp-1→, and β-D-Fru-(2→ was found to be 1:1:1. Therefore, the ratio of the three types of glycosidic bonds was 3.5:1:1.4. Methylation analysis revealed the ratio of →4)-α-D-Glcp-(1→, →4,6)-α-D-Glcp-(1→, β-D- Fru-(2→, and α-D-Glcp-1→ to be 19:4:8:1.

**Figure 4:**
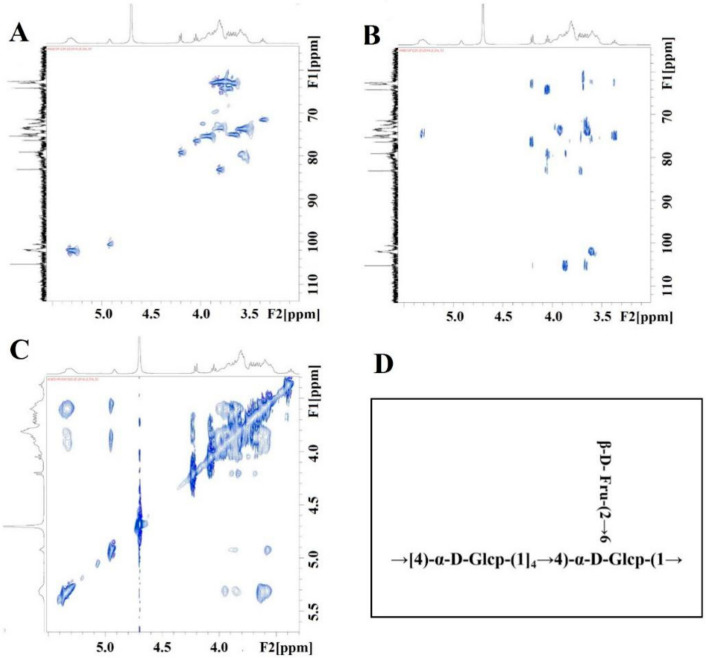
(**A**) HSQC; (**B**) HMBC; (**C**) NOESY spectra of SQWP-2; (**D**) Constitutional formula of SQWP-2.

The 2D spectra of SQWP-2 are shown in Figure 4A–C, respectively. HSQC spectrum revealed signal of the hetero-head carbon to be δ 5.31, the corresponding hetero-head hydrogen signal to be delta 5.31, and the signal H1-2 to be 5.31/3.55. The signal of H2-3 was 3.55/3.94 and that of H3-4 was 3.94/3.57. We can infer that H1, H2, H3, and H4 are 5.32, 3.55, 3.94 and 3.57 respectively, and the corresponding C5 was 73.53. δ 62.63 for C6 and δ 3.76 for H6a. Therefore, the signal can be attributed to the →4)-α-Glcp-(1→ glycosidic linkage. In addition, we observed the 105.17 ppm signal peak in the HSQC spectrum. There was no corresponding H, so we can infer that the signal relates to C2 of the Fru-2→ glycosidic linkage. We attributed this to the hydrocarbon Fru-2→ owing to the peak shape. We classified all glycosidic linkages according to the similarity rule combined with HMBC and NOESY results (Figure 4A,C; Table 3).

Glycoside linkage signals of polysaccharides were assigned from HMBC according to the 1D-2D NMR spectra. The δ 101.14 of the →4)-α-Glcp-(1→ glycoside linkage exhibited a signal peak corresponding with its H4 δ3.55, evidencing the existence of a →4)-α-D-Glcp-(1→4)-α-D-Glcp-(1→ linkage. The terminal α-D-Glcp-(1→ and Fru-(2→ group was found to be bonded to the main chain by *O*-6. The structural formula of SQWP-2 was shown in Figure 3G.

### 3.6. Cell Viability Analysis

**Figure 5:**
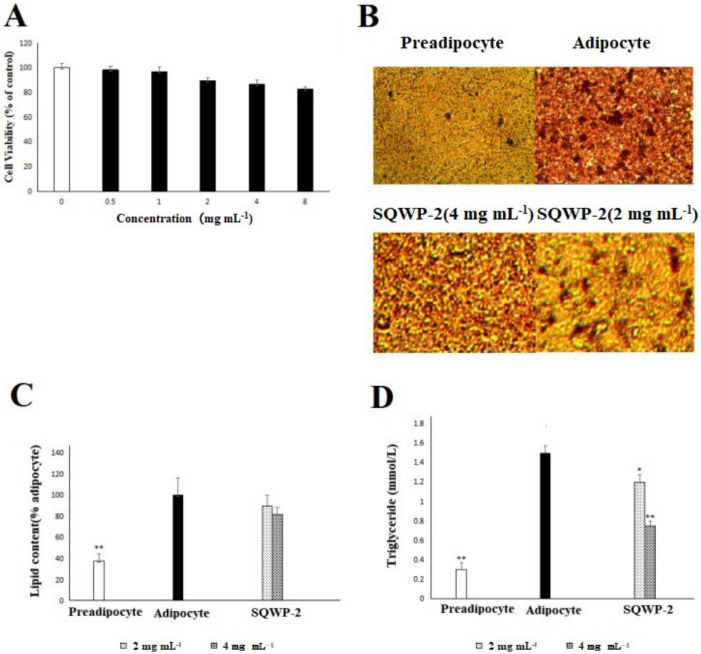
MTT cell viability of SQWP-2. Data are shown as mean ± SD. * *p* < 0.05 vs. control; ** *p* < 0.005 vs. control. (**A**) Effect of SQWP-2 on intracellular lipid accumulation in 3T3-L1 cells. Cells were treated with SQWP-2 (2 mg mL^−1^ and 4 mg mL^−1^) for day 8; (**B**) The mature adipocytes were colored with oil-red O; (**C**) OD value (% adipocyte); (**D**) TG content (mmol L^−1^) were measured to quantify intracellular lipid content.

There were no significant differences in the viability of 3T3-L1 cells exposed to 8 mg mL^−1^ SQWP-2 compared with the control group (Figure 5A). At the maximum concentration of SQWP-2 (8 mg mL^−1^), a 17.1% decrease in viability was observed, suggesting that SQWP-2 did not exert a significant toxic effect on 3T3-L1 cells at this concentration. When the SQWP-2 concentration was 4 mg mL^−1^, its toxic effect on cells could be ruled out. Therefore, a concentration of 4 mg mL^−1^ of SQWP-2 was used for further experiments.

### 3.7. Effect of the Water-Soluble Polysaccharide on Intracellular Lipid Accumulation in 3T3-L1 Cells

Oil-red O staining images showed that lipid accumulation was suppressed in the experimental group with SQWP-2 (Figure 4B). Lipid content was significantly decreased in the group treated with SQWP-2 (*p* < 0.05, Figure 4C). These results were confirmed by TG content (Figure 4D), TG level in SQWP-2-treated (4 mg mL^−1^) group was reduced by 49.7% compared with the adipocyte group (*p* < 0.05). From the effect of SQWP-2 on 3T3-L1 intracellular lipid accumulation, obviously, SQWP-2 with a concentration of 4 mg mL^−1^ was significantly better than 2 mg mL^−1^, so the concentration of SQWP-2 in subsequent experiments was 4 mg mL^−1^.

### 3.8. Effect of the Water-Soluble Polysaccharide on mRNA and Protein Expression

**Figure 6:**
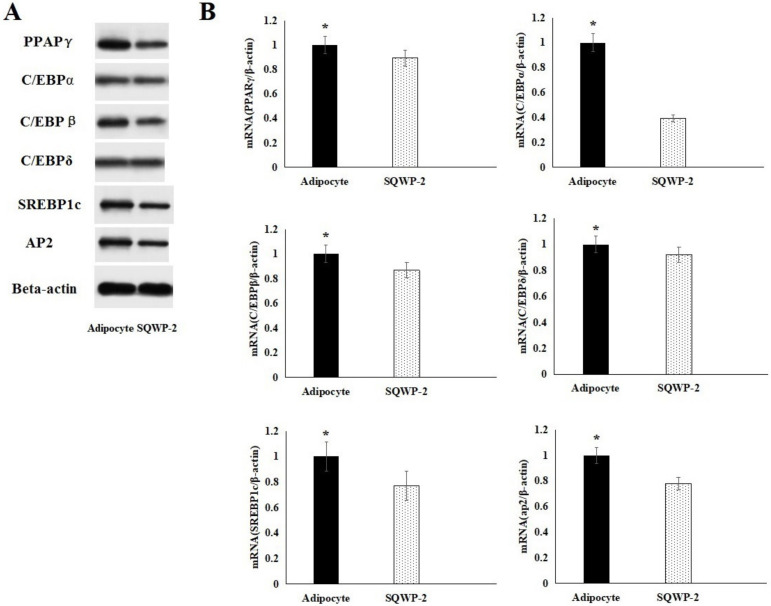
Effect of SQWP-2 on the protein levels (**A**) and mRNA expression (**B**) of related pathways in 3T3-L1 cells. Experiments were performed three times, and data are shown as mean ± SD. Values that do not share the same letter are significantly different (* *p* < 0.05).

Western Blotting revealed that protein expression reflected mRNA expression (Figure 6). The results suggested that SQWP-2 suppressed protein expression of PPARγ, C/EBPα, C/EBPβ, C/EBPδ, SREBP1C and AP2 by downregulating mRNA transcription. The expression levels of PPARγ, C/EBPα, C/EBPβ, C/EBPδ, SREBP1C and AP2 after treatment by SQWP-2 were reduced to 0.90 ± 0.06, 0.39 ± 0.03, 0.86 ± 0.06, 0.92 ± 0.06, 0.77 ± 0.05 and 0.78 ± 0.05 times of the control group, respectively (Figure 6B), which was consistent with the result of Western Blotting. SQWP-2 also be uncovered to suppress the mRNA expression of PPARγ, C/EBPα, C/EBPβ, C/EBPδ, SREBP1C and AP2 compared with control adipocytes. These major transcription factors work together to restrain adipocyte differentiation. The amplification curve and dissolution curve were shown in Figure S2.

## 4. Discussion

Polysaccharides are the main component of grains and have been reported to possess various bioactivities. Cordeiro et al. claimed that a linear arabinan with (1 → 5)-linked α-l-arabinofuranosyl units of polysaccharides in quinoa showed the strongest gastroprotective activity [25]. Quinoa polysaccharide constituted of Glc and Ara with a molar ratio of 1.17:1 was proven to prevent and protect against hyperlipidaemia [26]. Quinoa is becoming popular owing to the high nutritional value of the seeds. Our previous experiments have suggested the adipogenesis inhibitory effects of crude water-soluble polysaccharides extracts from quinoa. In the present study, to identify the responsible bioactive component, two polysaccharides fractions were separated and purified from crude water-soluble polysaccharides extracts. The SQWP-2 showed a higher inhibitory effect on 3T3-L1 cells differentiation than SQWP-1 group. Structural characterization of the polysaccharide revealed SQWP-2 had an average molecular weight of 7.49 × 10^3^ Da and consisted mainly of mannose and glucose with a ratio of 5.1:94.9. It is worth noting that fructose is a ketose, which can isomerize to mannose and glucose in a ratio of 52:48 during reduction. Galacturonic acid and glucose monosaccharides have been identified in a polysaccharide fraction purified from quinoa [15]. Three Astragalus polysaccharides prepared using different temperature treatments were found to have different chemical structures [27].

It is noteworthy that the present work is the first study to characterize glycosidic linkages using NMR spectroscopy including 1D and 2D spectra for this particular polysaccharide. The composition was found to comprise glucose, mainly dextran with a small amount of mannose. In the 1H-NMR spectrum, the prominent peak in the anomeric region indicated the presence of heterophase in Glc (p). The ^13^C NMR spectrum revealed several abnormal carbons in the pyranosyl residues and multiple non-anomalous carbon peaks within a wide area [28]. The linkage and NMR data suggest that →4)-α-Glc(p)-(1→ is the main connection unit in SQWP-2, similar to a polysaccharide isolated from Terminalia chebula [29]. Before this study, Cordeiro extracted the polysaccharides in quinoa and analyzed the structure and its gastroprotective activity [25]. The structure of quinoa polysaccharide in the study consisted of a linear arabinan with (1 → 5)-linked a-L-arabinofuranosyl units, which was different from results in this study. Two main reasons could contribute to this phenomenon. First, the source and variety of materials are different. Another is the different extraction method, 10% KOH was added in extracting polysaccharide. Due to these reasons, structural analysis and biological activity could show the difference. Research on enzymolysis and amylopectin chain-length distribution needs to be further studied to clarify the specific structural details of SQWP-2. For accuracy, we used the integral value of the proton hydrogen of each glycosidic linkage in the hydrogen spectrum to calculate the proportion of glycosidic bonds, as this exhibited good resolution without the need for processing. The glycosidic linkage →4,6) -α-D-Glcp-(1→ proton coincides with Glcp-1→. The ratio of glycosidic linkages was obtained through a methylation analysis of glycosidic linkage molar ratios.

As a nutritional supplement, quinoa has been reported to have anti-obesity activities and to have utility in the prevention and treatment of obesity [30]. Previous research has focused on the mechanism underlying the effects of quinoa in terms of increasing satiety, inhibiting digestive enzymes, and improving bacterial flora. Direct inhibition of adipocyte differentiation is another mechanism thought to contribute to the anti-obesity effects of quinoa. Polysaccharides are active compounds in many plants. In the present study, the isolated polysaccharide SQWP-2 strongly inhibited 3T3-L1 adipocyte differentiation. This can, therefore, be concluded to be one of the mechanisms explaining the anti-obesity activity of quinoa, which is a novel finding because the contribution of fullness to the anti-obesity effect has been the focus of previous research into quinoa. To further study the mechanism of SQWP-2 inhibition of differentiation of 3T3-L1 adipocytes, we studied the mRNA and protein expression of related protein factors. The protein PPARγ is an essential factor in adipocyte differentiation, while the expression of C/EBPα remains constant. This is similar to previous research results, which have shown that the morus polysaccharide effectively inhibits adipocyte differentiation [1]. The expression of SREBP1C has been reported to be similar to that of C/EBPβ, which is consistent with the results of this study. We also found that SQWP-2 strongly inhibited C/EBPα expression which supports previous research [31]. The transcription factor C/EBPα coordinates cell differentiation and growth arrest [32,33].

Polysaccharides with different structures have different biological activities. For example, the immunomodulatory activities of polysaccharides are reported to be influenced by the monosaccharides and glycosidic linkage compositions [34]. Toll-like receptor 4, an important receptor of immunomodulatory activity, is closely associated with high-glucose polysaccharides such as β-(1,3)-Glc, β-(1,4)-Glc, and α-(1,4)-Glc. The main chain of polysaccharide from fungus has been reported to be composed of →3,6)-β-L-Man-(1→, α-D-Glc-(1→, →4)-α-D-Glc-(1→, →3,6)-β-D-Gal-(1→, and →6)-β-D-Gal-(1→, and the polysaccharide exhibits strong immunomodulatory activity [35]. Many plant polysaccharides have been demonstrated to inhibit 3T3-L1 adipocyte differentiation [12], and quinoa polysaccharides have been confirmed to have immunomodulatory functions [18]. However, the structural details and anti-obesity activity of quinoa polysaccharides are poorly studied to date. This inhibitory effect has also been demonstrated for polysaccharides isolated from Pine needles [35], Gray Oyster mushrooms [36], and Nannochloropsis oculata [33], and were shown to be positively correlated with molecular weight. The anti-obesity activity of polysaccharides largely depends on their structure and the intracellular signaling pathways involved. Fucoidan has been reported to inhibit the expression of the early C/EBPα and PPARγ and the late AP2 adipose-forming transcription factors, which are crucial for the development of fat cells [31]. The present research found the presence of →4)-α-D-Glcp-(1→ glycoside linkages with α-D-Glcp-(1→ and Fru-(2→ to result in strong inhibition of 3T3-L1 adipocyte differentiation. Our study elucidated the mechanism of the inhibitory effects of SQWP-2 on 3T3-L1 adipocyte differentiation.

## 5. Conclusions

The isolated polysaccharide was found to consist of fructose and glucose in a ratio of 9.8:90.2 with an average molecular weight of 7.49 × 10^3^ Da. The main sugar residue linkages were found to be →4)-α-D-Glcp-(1→: →4,6)-α-D-Glcp-(1→: β-D-Fru-(2→: α-D-Glcp-1→, existing at a ratio of close to 19:4:8:1. The main chain connection mode of the polysaccharide was determined to be a →4)-α-D-Glcp-(1→ glycoside linkage, while the terminal group of α-D-Glcp-(1→ and Fru-(2→ was bonded to the main chain via *O*-6. The polysaccharide inhibited 3T3-L1 adipocyte differentiation, indicating that proliferation was inhibited through the promotion of PPARγ, C/EBPα C/EBPβ, C/EBPδ, SREBP1C and AP2 expression. The expression levels were reduced to 0.90 ± 0.06, 0.39 ± 0.03, 0.86 ± 0.06 and 0.92 ± 0.06, 0.77 ± 0.05, 0.78 ± 0.05 times of the control group, respectively. Based on the above result, SQWP-2, as an active ingredient food, has broad application prospects due to its inhibitory effects on 3T3-L1 adipocyte differentiation.

## Figures and Tables

**Table 1:** The primer sequence is used for real-time polymerase chain reaction (PCR).

Gene Name	Forward Primer	Reverse Primer	Accession No.
C/EBPα	TTACAACAGGCCAGGTTTCC	GGCTGGCGACATACAGTACA	NM_007678
PPARγ	TTTTCAAGGGTGCCAGTTTC	AATCCTTGGCCCTCTGAGAT	NM_011146
C/EBPβ	CCTTTAAATCCATGGAAGTGG	GGGCTGAAGTCGATGGC	NM_005194.2
C/EBPδ	ACGACGAGAGCGCCATC	TCGCCGTCGCCCCAGTC	TRCN0000013697
AP2	GGCCAAGCCCAACATGATC	CACGCCCAGTTTGAAGGAAA	NM_024406
M-SREBP1c	ACAGACAAACTGCCCATCCA	GCAAGAAGCGGATGTAGTCG	NC_010454.4
β-actin	CCACAGCTGAGAGGGAAATC	AAGGAAGGCTGGAAAAGAGC	X03672

**Table 2:** Three glycoside bonds of quinoa polysaccharide.

Methylated Sugar	Mass Fragments (*m*/*z*)	Type of Linkage
2,3,4,6-Me4-Glcp	43,71,87,101,117,129,145,161,205	Glc-(1→
1,3,4,6-Me4-Glc/Manp	87,101,129,145,161	Fru-(2→
2,3,6-Me3-Glcp	43,87,99,101,113,117,129,131,161,173,233	→4)-Glcp-(1→
2,3-Me2-Glcp	43,71,85,87,99,101,117,127,159,161,201	→4,6)-Glcp-(1→

**Table 3:** The attribution of Hydrocarbon signal.

Glycosyl Residues	H1	H2	H3	H4	H5	H6a	H6b
	C1	C2	C3	C4	C5	C6	
→4)-α-D-Glcp-(1→	5.31	3.55	3.94	3.57	3.78	3.76	ns
	102.01	73.57	75.31	79.58	73.52	62.63	
→4,6)-α-D-Glcp-(1→	4.93	3.55	3.65	3.54	3.85	3.84	ns
	100.7	73.53	74.02	78.2	74.6	69.71	
Fru-(2→	3.65/3.81		4.2	4.05	3.81	3.72/3.78	
	62.74	105.17	79.01	76.4	83.23	64.28	
α-D-Glcp-1→	5.26	3.36	3.66	3.95	3.98	3.6	3.82
	101.33	71.4	75.1	70.61	72.41	62.3	

## Supplementary Materials

The following are available online at https://www.mdpi.com/2304-8158/9/10/1511/s1, Figure S1: Effects of SQWP-1 and SQWP-2 on 3T3-L1 cells inhibiting differentiation., Figure S2: Amplification curve and Dissolution curve of β-actin, PPARγ, C/EBPα, C/EBPβ, C/EBPδ, SREBP1C, AP2.

## Abbreviations

PPARγ	Peroxisome proliferator-activated receptor gamma
C/EBPα	CCAAT/enhancer-binding protein alpha
C/EBPβ	CCAAT/enhancer-binding protein beta
C/EBPδ	CCAAT/enhancer-binding protein delta
AP2	Adipocyte protein 2
M-SREBP1C	Sterol regulatory element-binding protein-1c
Dept	Distortionless Enhancement Polarizationtransfer
HSQC	Heteronuclear Single Quantum Coherence
HMBC	Heteronuclear Multiple Bond Correlation
NOESY	Nuclear Overhauser Effect Spectroscopy
TG	Triglyceride
