# Supplementary material for: Structural Characterization of Quinoa Polysaccharide and Its Inhibitory Effects on 3T3-L1 Adipocyte Differentiation
Source: Foods. 2020 Oct 21;9(10):1511. doi: 10.3390/foods9101511 (PMC7589720; doi:10.3390/foods9101511)
Supplement: Supplementary file 1 [file foods-09-01511-s001.pdf]

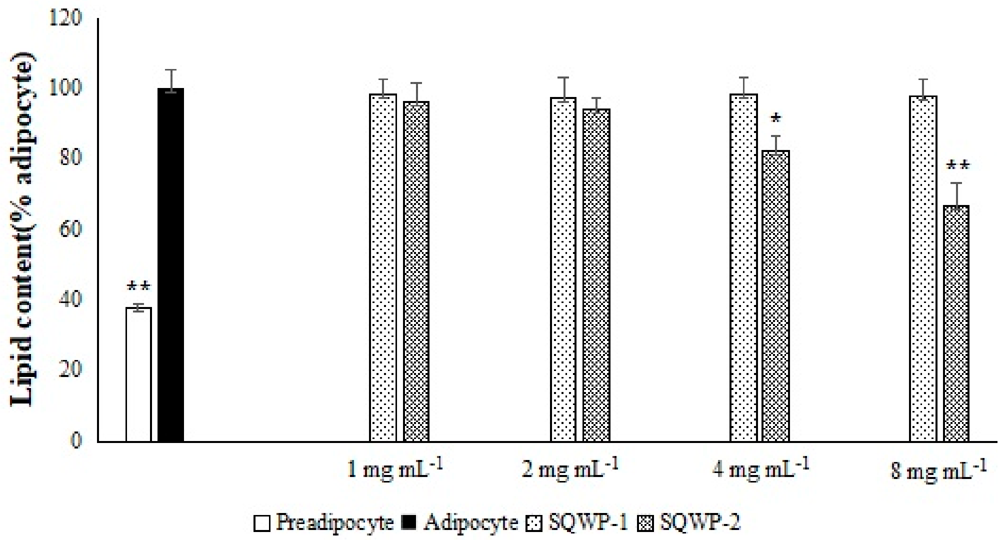

Figure S1. Effects of SQWP-1 and SQWP-2 on 3T3-L1 cells inhibiting differentiation.

β-actin: Amplification curve

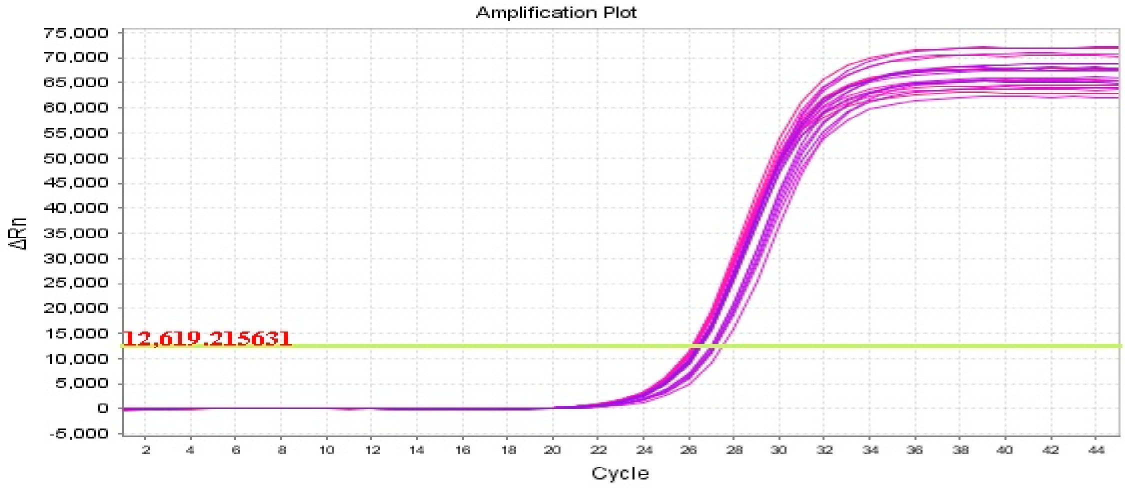

Dissolution curve

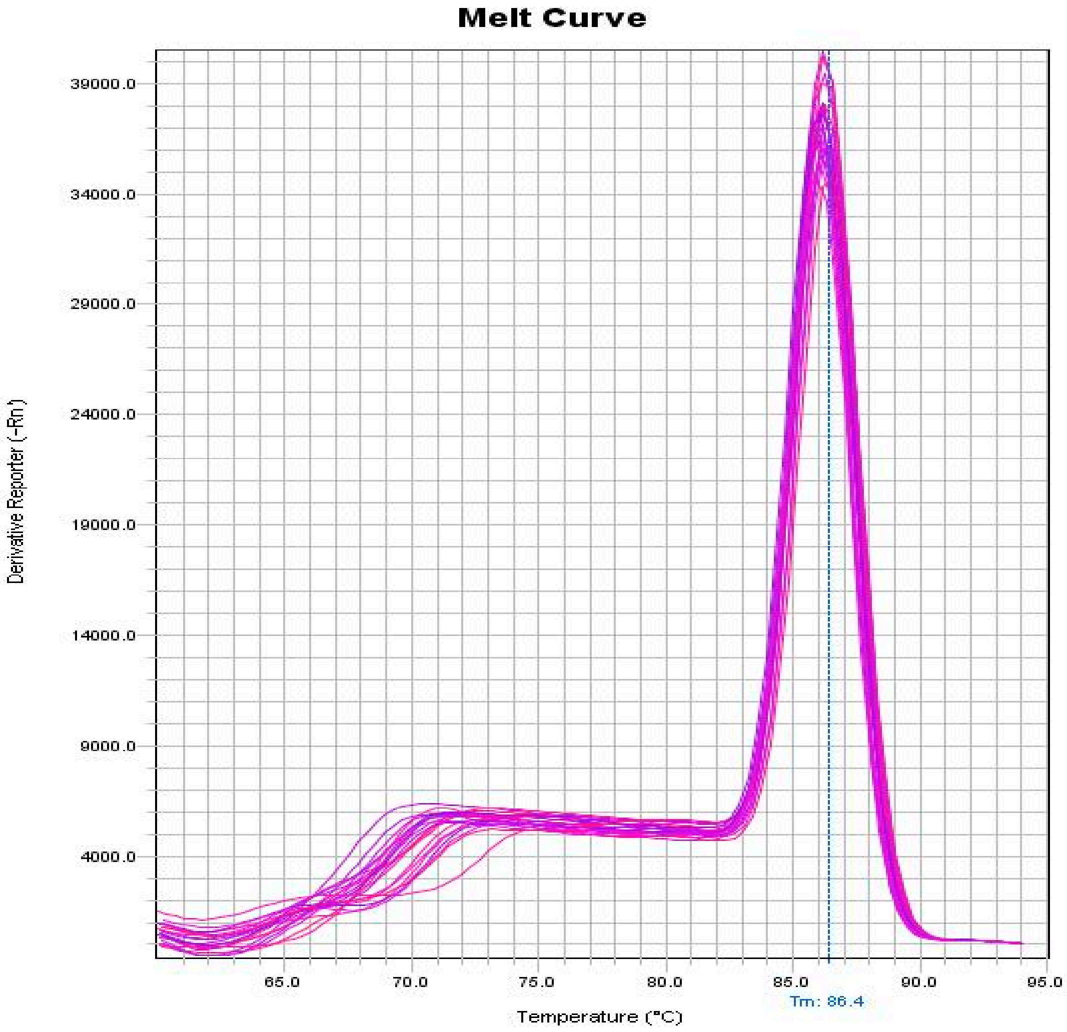

PPARγ: Amplification curve

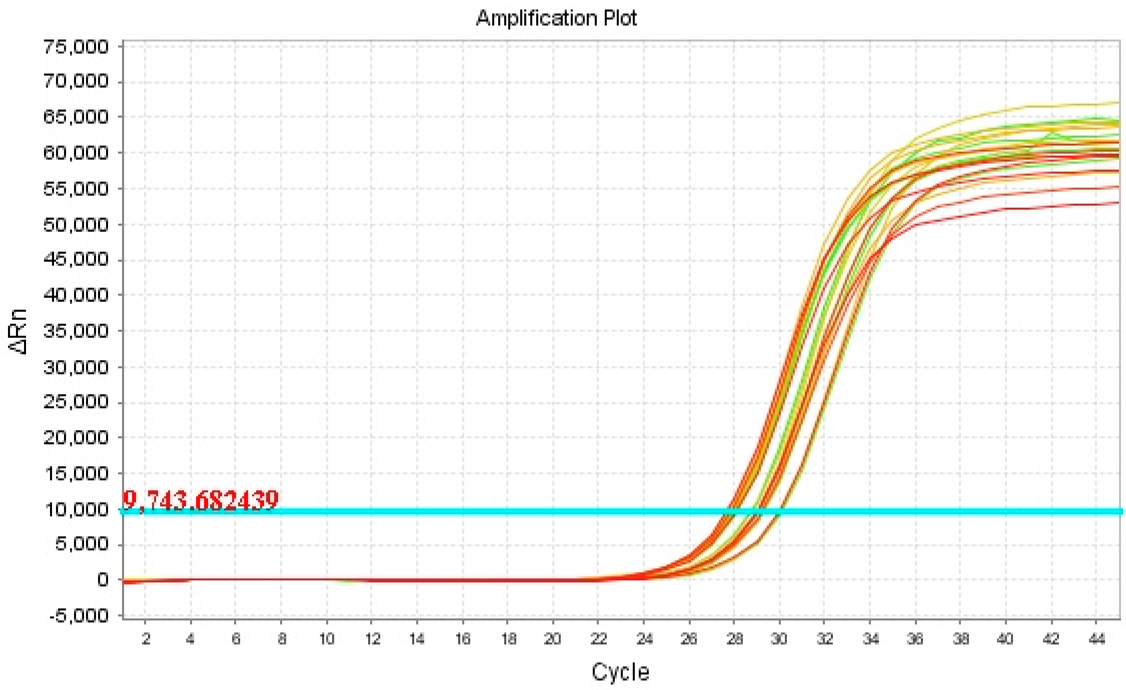

Dissolution curve

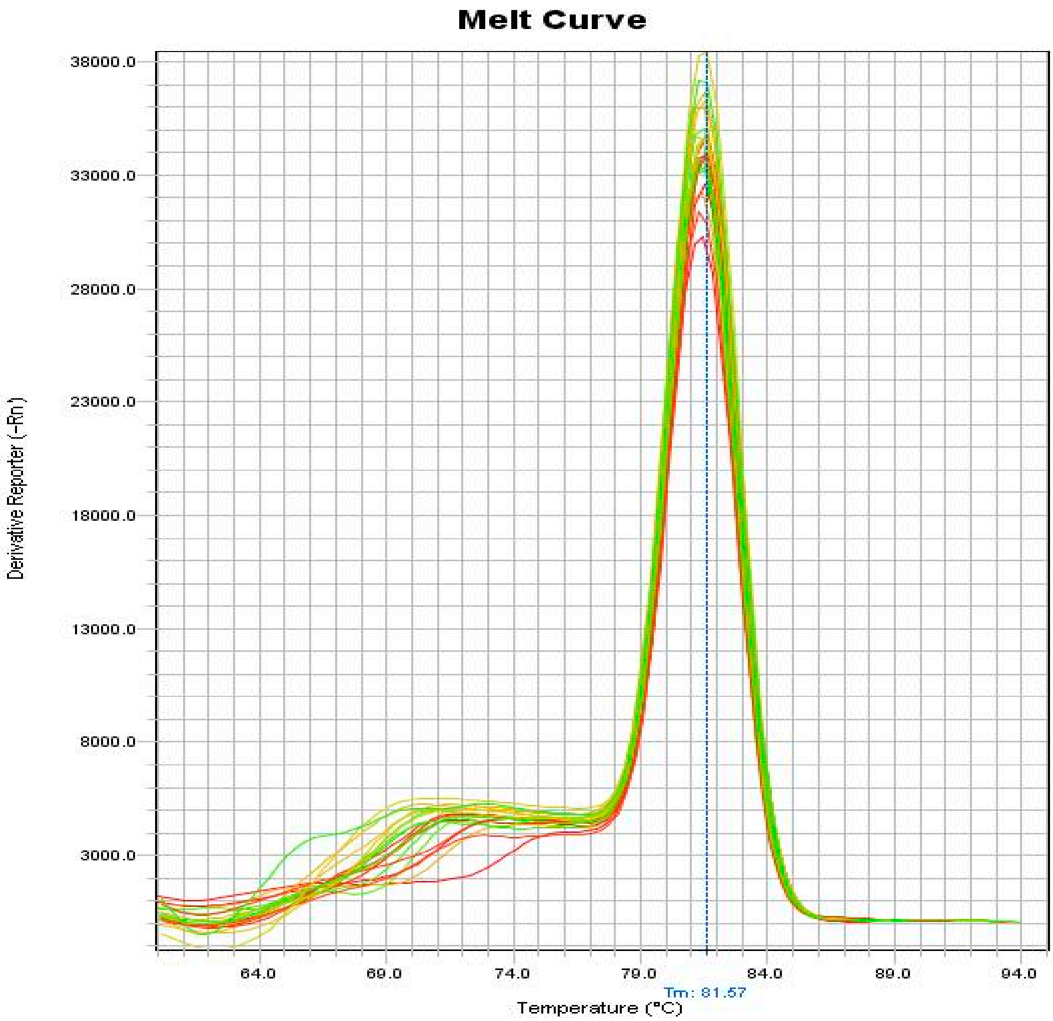

C/EBPα: Amplification curve

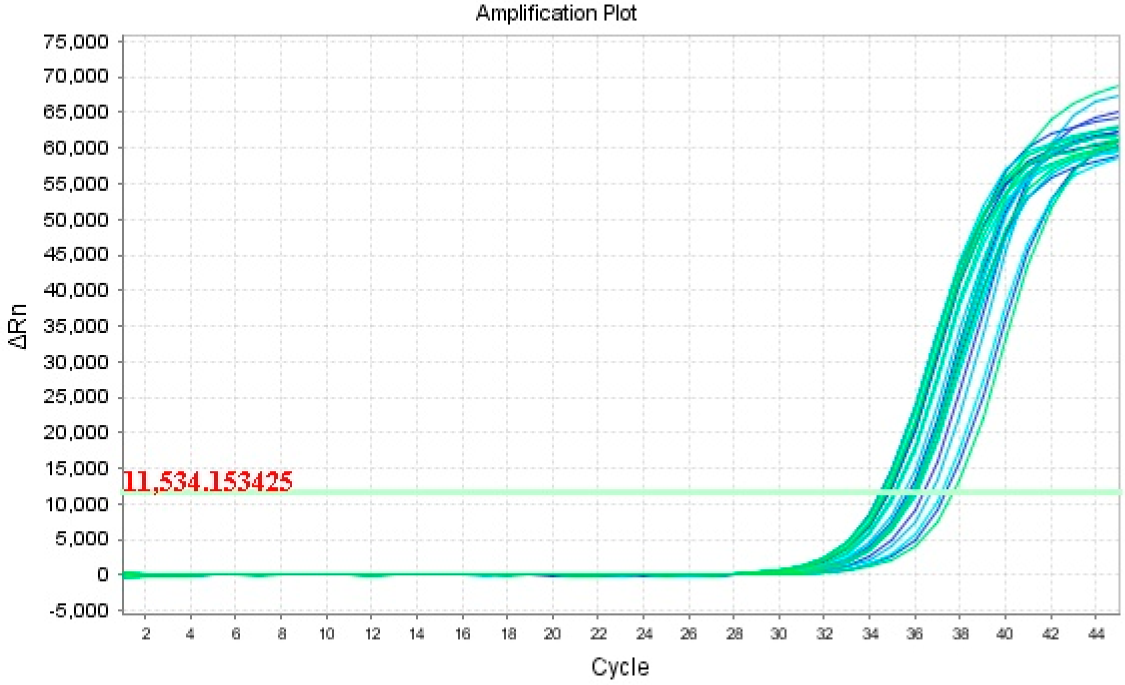

Dissolution curve

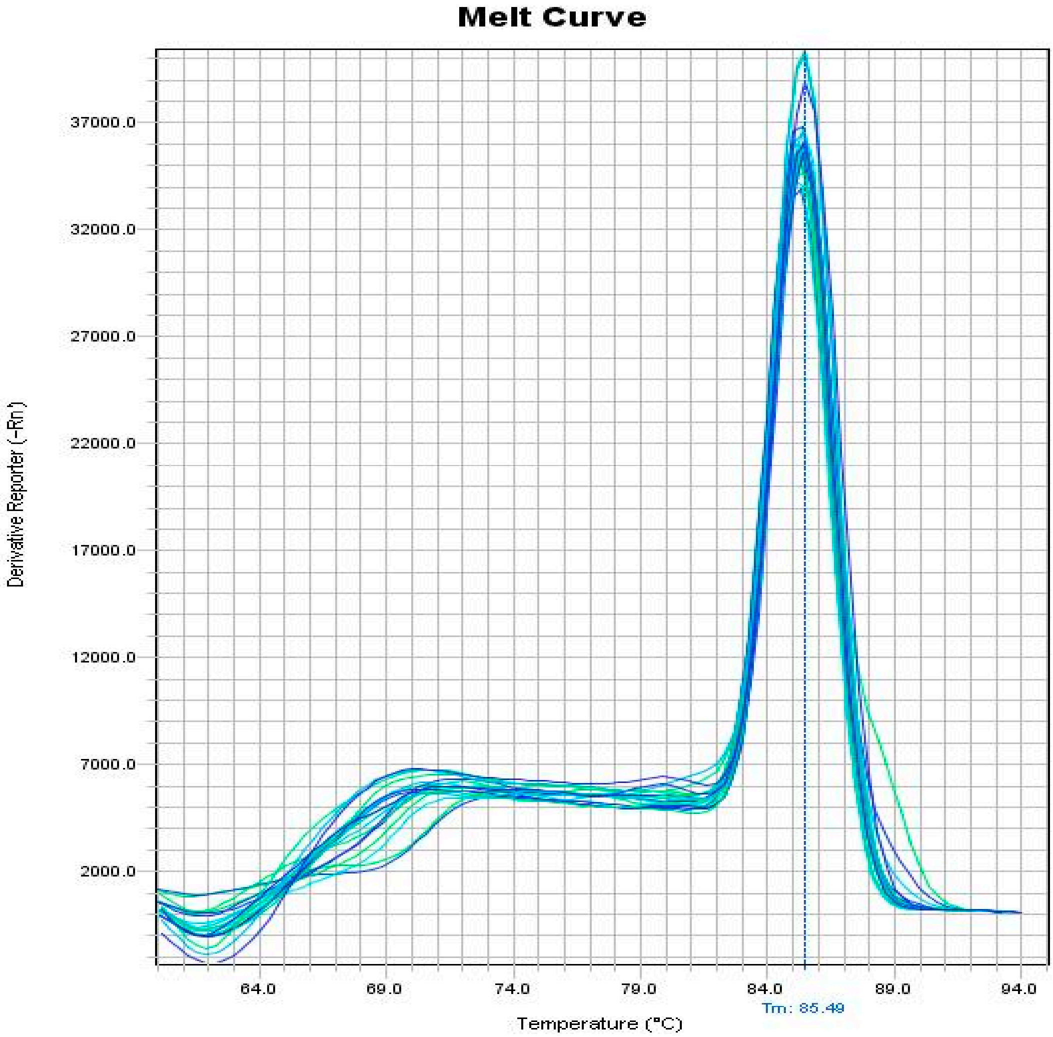

C/EBPβ: Amplification curve

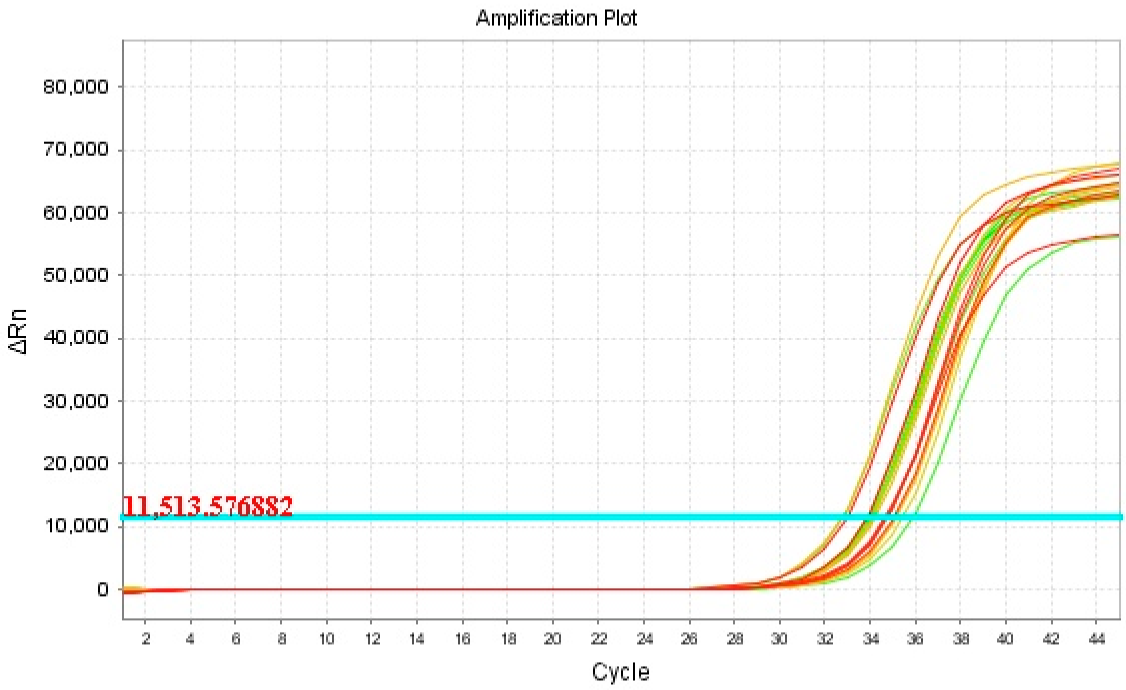

Dissolution curve

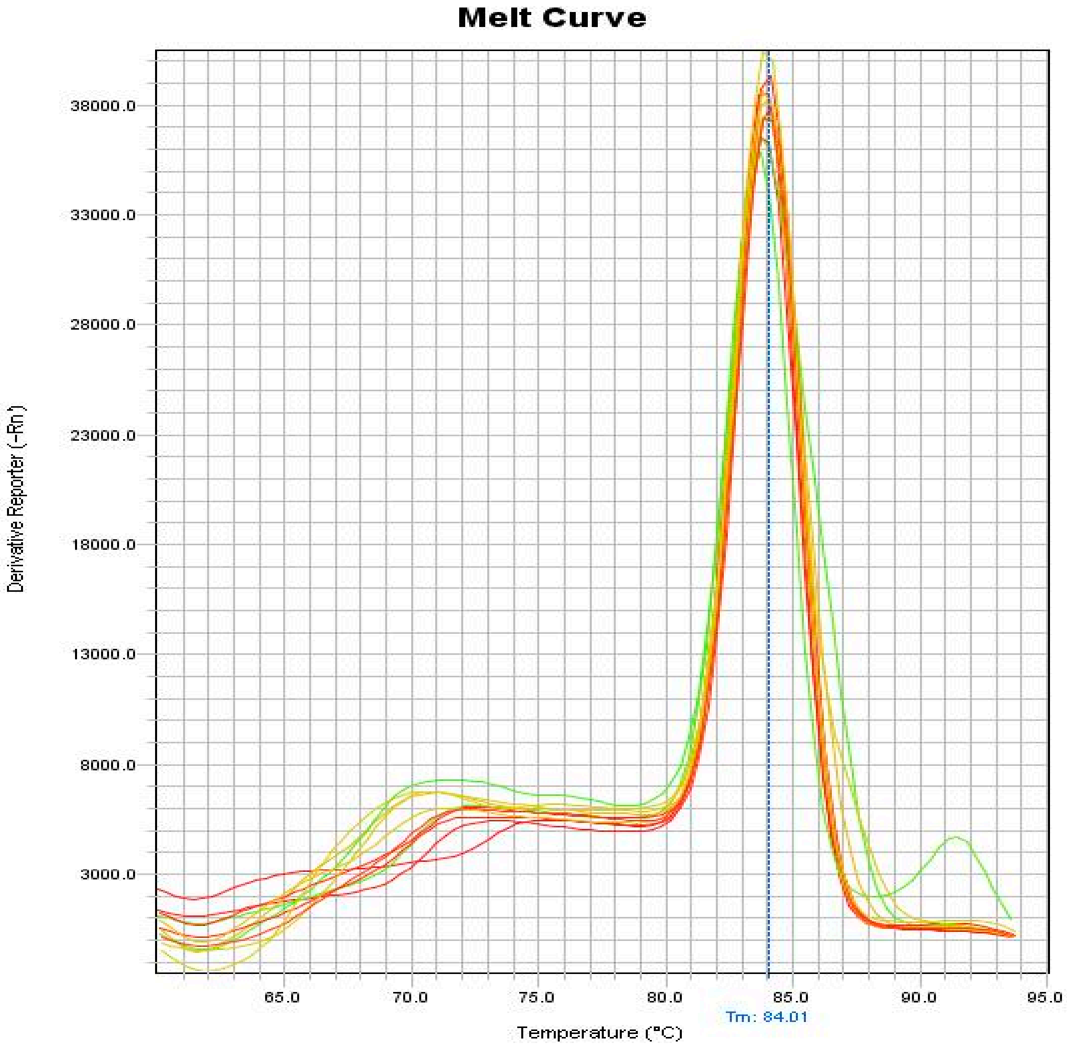

C/EBPδ: Amplification curve

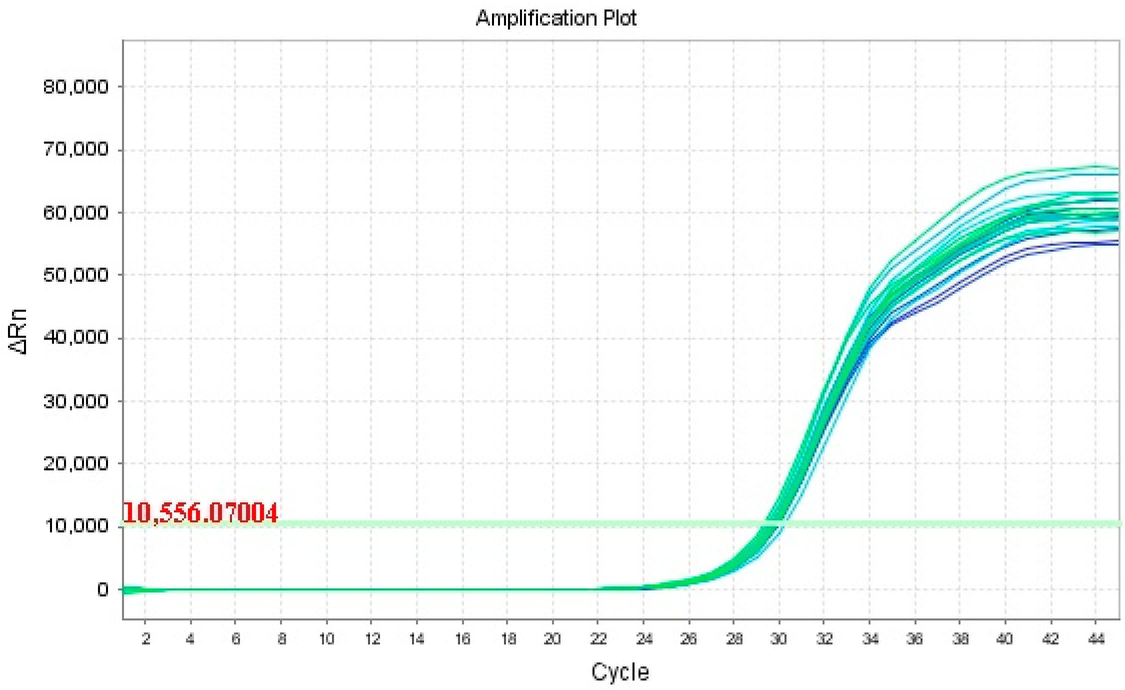

Dissolution curve

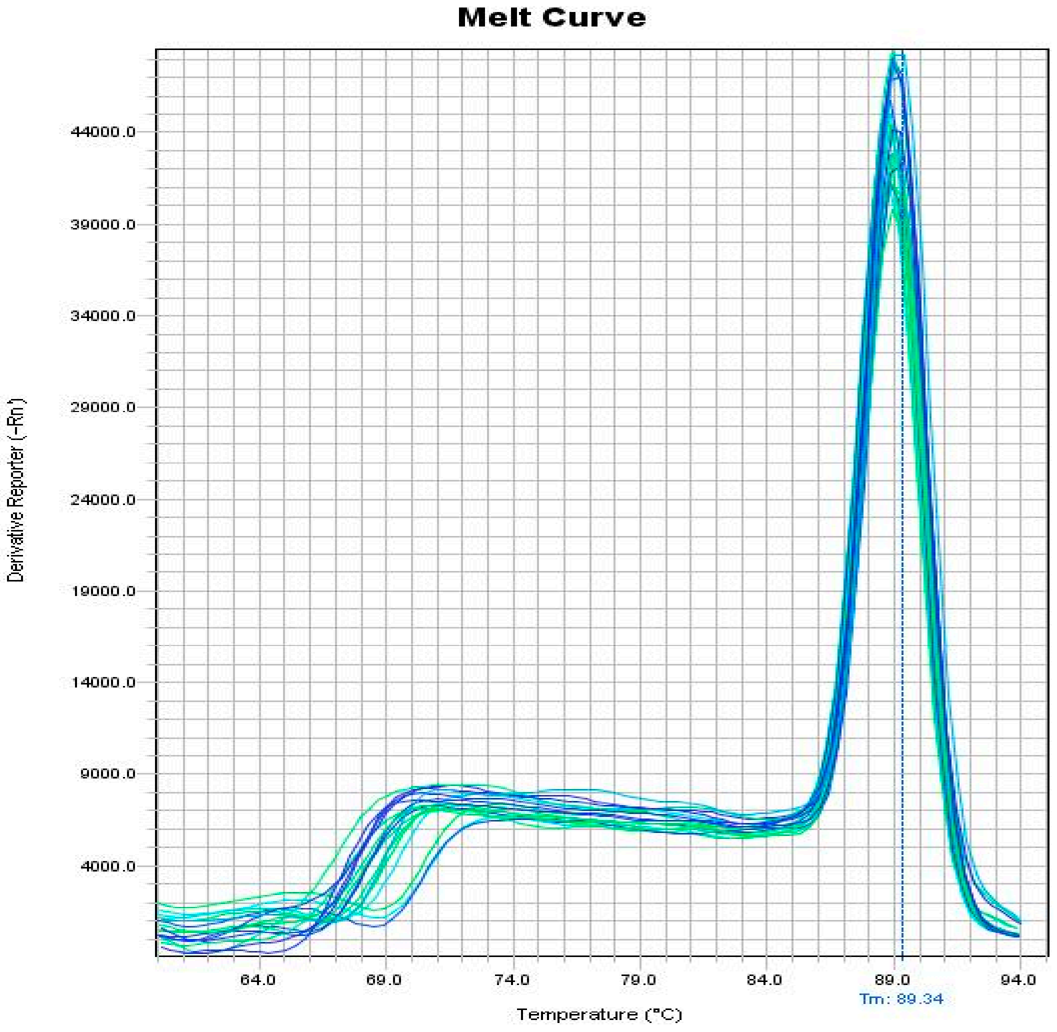

SREBP1c: Amplification curve

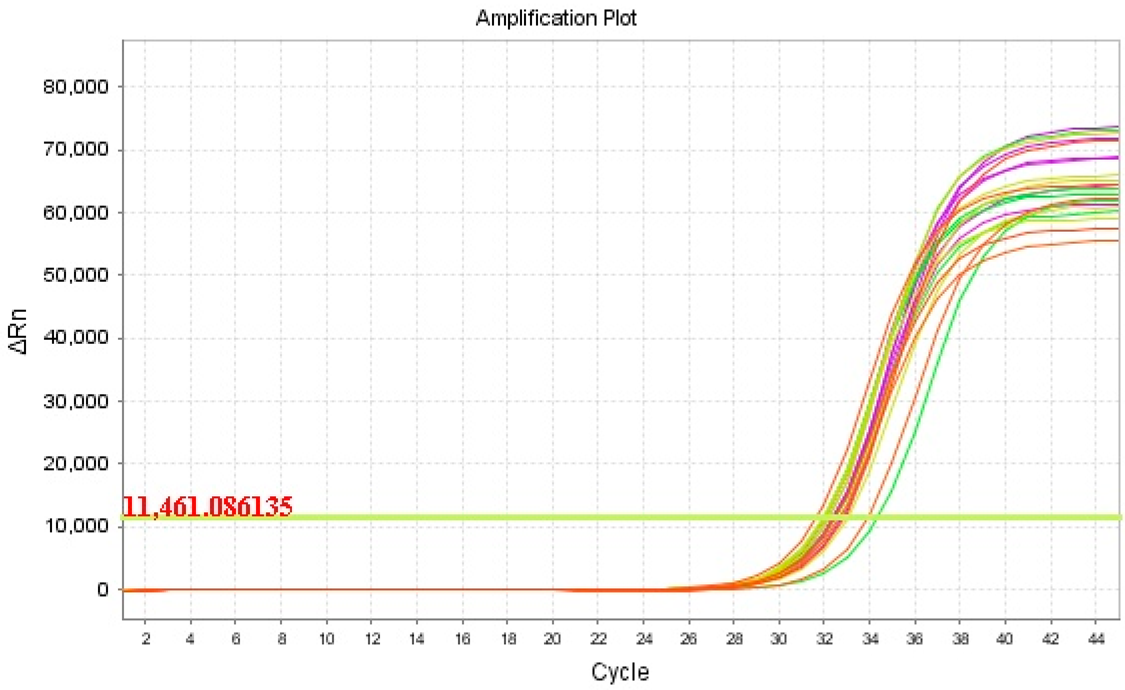

Dissolution curve

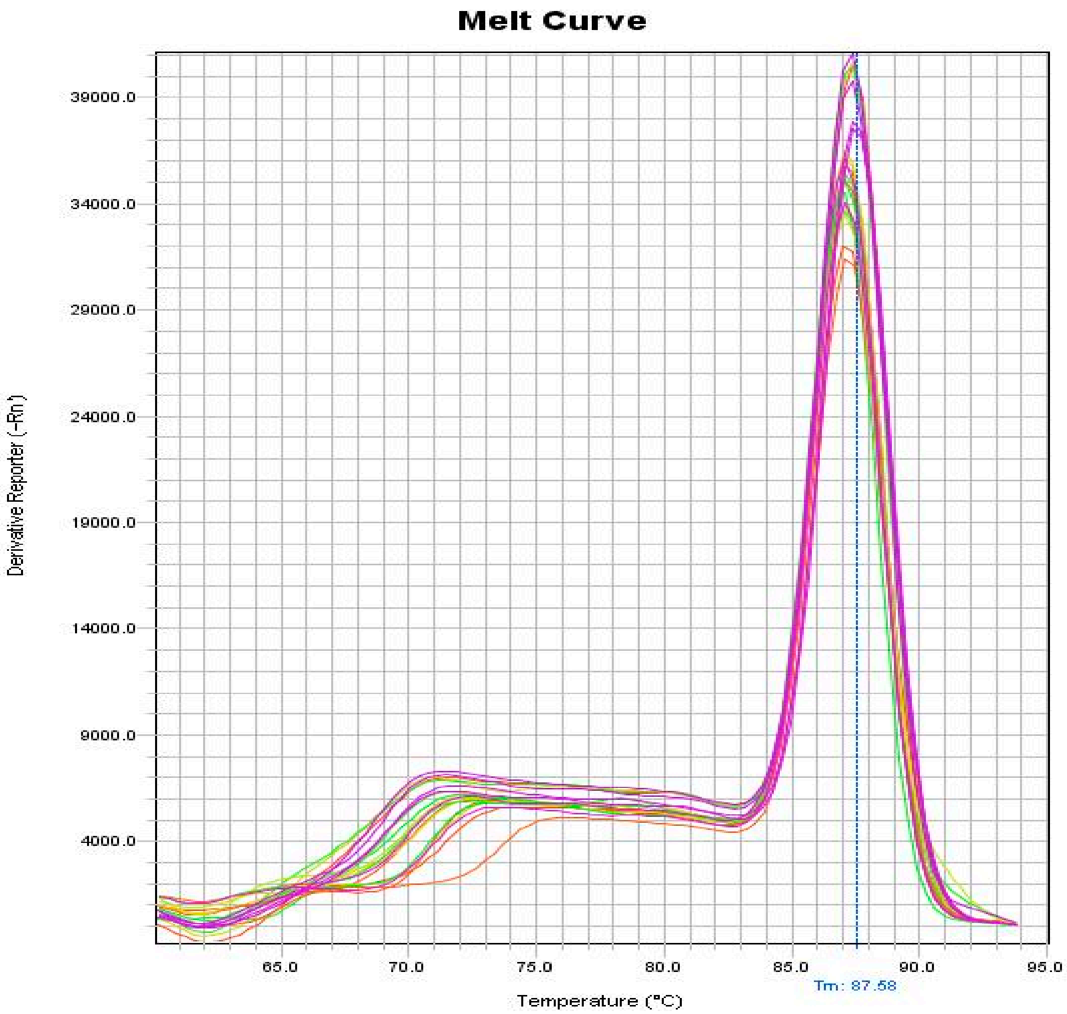

AP2: Amplification curve

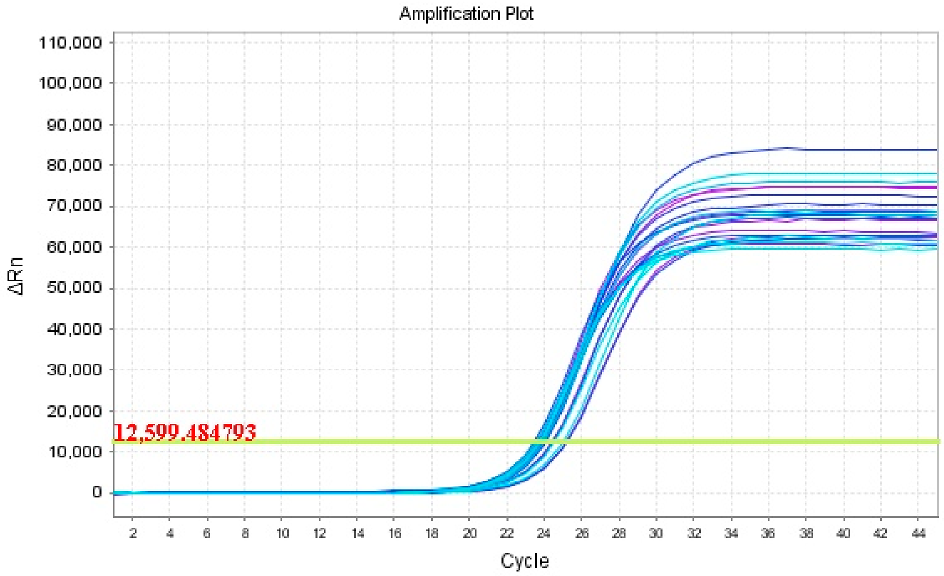

Dissolution curve

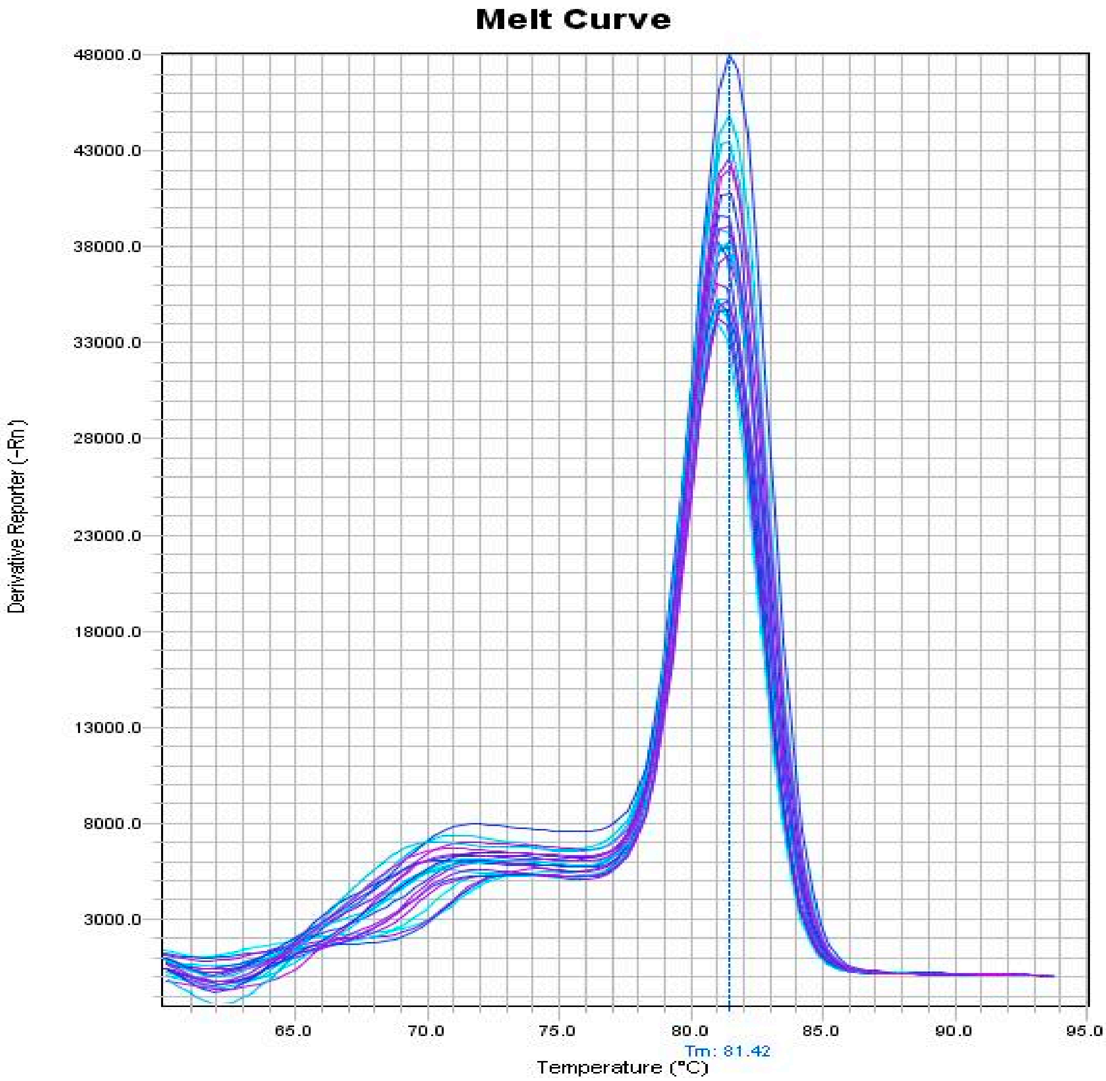

Figure S2. Amplification curve and Dissolution curve of β-actin, PPARγ, C/EBPα, C/EBPβ,

C/EBPδ, SREBP1C, AP2.
